# Tyrosine Kinase ETK/BMX Is Up-Regulated in Bladder Cancer and Predicts Poor Prognosis in Patients with Cystectomy

**DOI:** 10.1371/journal.pone.0017778

**Published:** 2011-03-07

**Authors:** Shengjie Guo, Feng Sun, Zhiyong Guo, Wei Li, Alan Alfano, Hegang Chen, Clara E. Magyar, Jiaoti Huang, Toby C. Chai, Shaopeng Qiu, Yun Qiu

**Affiliations:** 1 Department of Pharmacology and Experimental Therapeutics and The Greenebaum Cancer Center, University of Maryland School of Medicine, Baltimore, Maryland, United States of America; 2 Department of Epidemiology and Preventive Medicine, University of Maryland School of Medicine, Baltimore, Maryland, United States of America; 3 Division of Urology, University of Maryland School of Medicine, Baltimore, Maryland, United States of America; 4 Department of Urology, First Affiliated Hospital, Sun Yat-sen University, Guangzhou, China; 5 Department of Pathology and Laboratory Medicine, The David Geffen School of Medicine at University of California Los Angeles, Los Angeles, California, United States of America; University of Pennsylvania, United States of America

## Abstract

Deregulation of the non-receptor tyrosine kinase ETK/BMX has been reported in several solid tumors. In this report, we demonstrated that ETK expression is progressively increased during bladder cancer progression. We found that down-regulation of ETK in bladder cancer cells attenuated STAT3 and AKT activity whereas exogenous overexpression of ETK had opposite effects, suggesting that deregulation of ETK may attribute to the elevated activity of STAT3 and AKT frequently detected in bladder cancer. The survival, migration and invasion of bladder cancer cells were significantly compromised when ETK expression was knocked down by a specific shRNA. In addition, we showed that ETK localizes to mitochondria in bladder cancer cells through interacting with Bcl-XL and regulating ROS production and drug sensitivity. Therefore, ETK may play an important role in regulating survival, migration and invasion by modulating multiple signaling pathways in bladder cancer cells. Immunohistochemistry analysis on tissue microarrays containing 619 human bladder tissue samples shows that ETK is significantly upregulated during bladder cancer development and progression and ETK expression level predicts the survival rate of patients with cystectomy. Taken together, our results suggest that ETK may potentially serve as a new drug target for bladder cancer treatment as well as a biomarker which could be used to identify patients with higher mortality risk, who may be benefited from therapeutics targeting ETK activity.

## Introduction

Bladder cancer is one of the most common cancers in the United States. It is estimated that there will be 70,530 new cases and 14,680 deaths due to bladder cancer in 2010 [1], [2], [3]. Multiple genetic and epigenetic factors are believed to contribute to the risk of developing bladder cancer. In addition to well known risk factors such as aging, gender, exposure to cigarette smoke and industrial chemicals, several genetic lesions that provide cues for cell transformation, proliferation, migration and invasion in bladder cancer have been identified [4]. These include mutations or alterations in expression of p53, pRb, E-cadherin, COX2, BLCA-4, CXCL1, MMP-2/9 and EGFR [5]. Cumulative increase in these genetic defects also provides prognostic assessment for disease outcome. However, further understanding of bladder tumor biology is necessary to develop more effective therapy. There is thus a need for the identification and application of novel therapeutic targets in bladder cancer.

Epithelial and endothelial tyrosine kinase (ETK), also known as Bone Marrow X kinase (BMX), is a member of Tec family non-receptor tyrosine kinase. ETK contains an NH2-terminal Pleckstrin homology domain, a Src homology 3 domain, a Src homology 2 domain, and a COOH-terminal tyrosine kinase domain [6]. ETK can be activated by several extracellular stimuli, including growth factors, cytokines, extracellular matrix and hormones [7]. ETK protein is present in cytoplasm with strong perinuclear staining in cells when examined using immunofluorescence microscopy [8], [9], [10]. Activation of ETK kinase activity can be achieved by interaction of its pleckstrin homology domain either with the phosphatidylinositol 3-kinase product phosphatidylinositol 3,4,5-triphosphate or the FERM domain of FAK, which leads to plasma membrane translocation of ETK [6], [8]. ETK has been shown to play a role in various cellular processes including cell proliferation, transformation, differentiation, migration and metastasis. ETK can interact with FAK and p130^Cas^ to regulate actin cytoskeleton and cell motility [8], [11]. It has also been shown that ETK directly interacts with tumor suppress p53 and inhibit its nuclear translocation, thereby promoting chemoresistance in cancer cells [9]. We previously showed that ETK is progressively upregulated during human prostate cancer development and progression [12], [13]. However, the role of ETK in bladder cancer cells remains unknown.

In this report, we demonstrated that ETK expression is progressively increased during bladder cancer progression. ETK plays an important role in regulating survival, migration and invasion by modulating multiple signaling pathways in bladder cancer cells. We further showed that ETK is also located in mitochondria through directly interacting with Bcl-XL and regulating ROS production in response to treatment of chemotherapeutic drugs. Furthermore, ETK expression positively correlates tumor grade and predicts patient outcome. Our data suggest that ETK may potentially serve as a drug target and prognostic marker for bladder cancer.

## Results

### Functional Etk is overexpressed in bladder cancer

**Figure 1:**
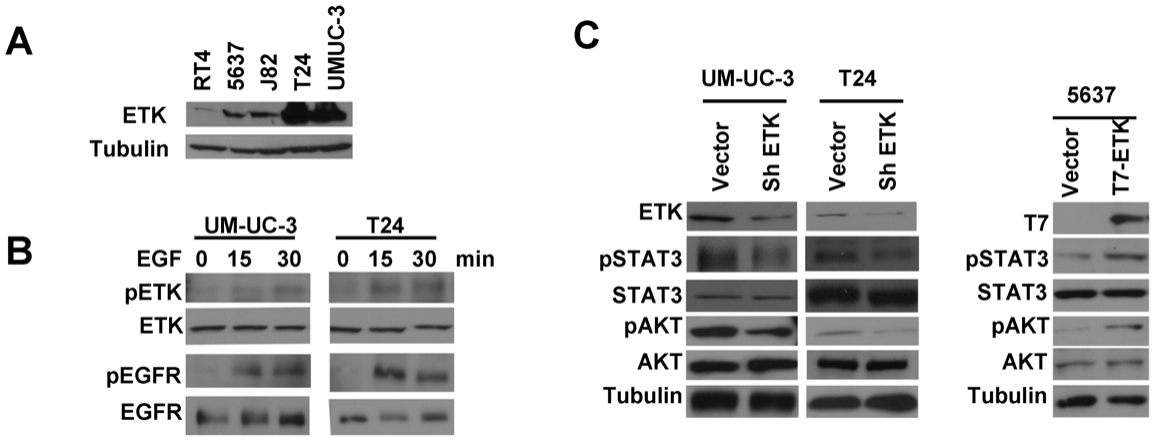
Functional Etk is expressed in bladder cancer cell lines. **A**, Expression profile of ETK in bladder cancer cell lines. ETK expression level in total cell lysates from bladder cancer cell lines was examined by immunoblotting with anti-ETK. Tubulin was used as a loading control. **B**, Activation of ETK in bladder cancer cells treated with EGF. UM-UC-3 and T24 cells were serum starved for 24 h, then treated with 20 ng/ml EGF for 15 or 30 min. The level of activated EGFR and ETK was monitored by immunoblotting with anti-pEGFR(Y1175) and anti-pETK(Y40) respectively. The level of total EGFR and ETK in the cell lysates was also detected with anti-EGFR and anti-ETK respectively. **C**, Regulation of STAT3 and AKT activity by ETK in bladder cancer cells. UM-UC-3 and T24 cells were infected with lentivirus encoding the shRNA specific for ETK or a vector control. At 24 h post-infection, cells were serum starved overnight and then lysed. The level of active STAT3 and AKT was examined by immunoblotting with anti-pSTAT3 (Y705) and anti-pAKT (S473) respectively (Left panel). The effect of ETK overexpression on STAT3 and AKT activity was also examined in bladder cancer 5637 cells (Right panel).

We examined ETK expression in a panel of human bladder cancer cell lines and found that ETK expression level was varied in these cells. Interestingly, a significantly higher level of ETK protein was detected in UM-UC-3 and T24 cells which are derived from high-grade and invasive bladder tumors (Fig. 1A). We then examined whether ETK is functional in these invasive cells. We first tested whether growth factor could activate ETK kinase activity using phosphorylation of tyrosine 40 (Y40), an autophosphorylation site by ETK upon its activation [8], as readout. As shown in Fig. 1B, EGF treatment induced Y40 phosphorylation, suggesting that ETK is active in bladder cancer cells. Consistent with previous studies showed that ETK is upstream of STAT3 and AKT pathways [15], [16], we found the phosphorylation level of both STAT3 and AKT was compromised when ETK expression was knocked down by a specific shRNA (Fig. 1C, Left). Conversely, when we overexpressed ETK in 5637 cells with a relatively lower level of endogenous ETK, we detected an increased activity of both STAT3 and AKT (Fig. 1C, Right). These data together indicated that ETK is highly expressed in invasive bladder cancer cell lines and involved in regulating STAT3 and AKT activity.

### Etk is present in mitochondria and associated with Bcl-XL

**Figure 2:**
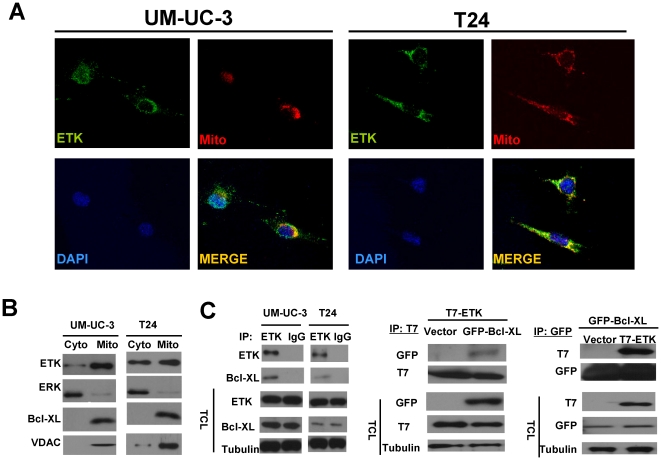
ETK localizes to mitochondria and interacts with Bcl-XL in bladder cancer cells. **A**, ETK localizes to mitochondria in UM-UC-3 and T24 cells. Cells were labeled with Rhodamine Mitotracker, followed by immunofluorescence staining with anti-ETK. Nuclei were counterstained using DAPI. Localization of ETK was determined by confocal microscopy. Yellow shows colocalization of ETK and Mitotracker. **B**, ETK protein is detected in mitochondrial fraction. Mitochondrial and cytosolic fractions were prepared as described in Methods. The fractionated extracts were subjected to immunoblotting with anti-ETK or indicated antibodies for fractionation markers. ERK was used as a cytosolic marker, while Bcl-XL and VDAC as mitochondrial markers. **C**, ETK is associated with Bcl-XL. Total cell extracts from UM-UC-3 and T24 cells were immunoprecipitated with anti-ETK or IgG control, followed by immunoblotting with the antibodies as indicated (Left panel). 293T cells were co-transfected with T7-Etk and GFP-Bcl-XL, immunoprecipitation was performed using anti-T7 (Center panel) or anti-GFP (Right panel), followed by immunoblotting with the antibodies as indicated.

**Figure 3:**
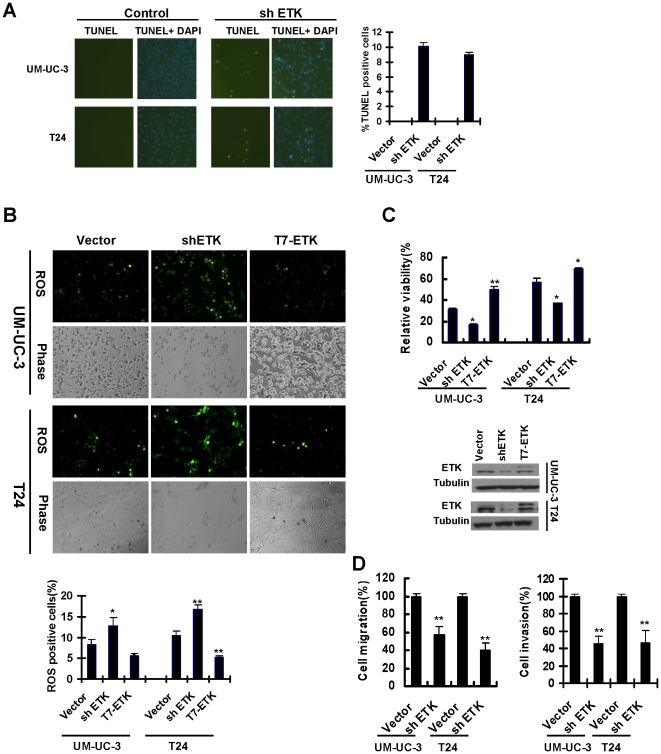
Regulation of survival, migration and invasion by ETK in bladder cancer cells. **A**, Down-regulation of ETK expression induced apoptosis in bladder cancer cells. UM-UC-3 and T24 cells were infected with lentivirus encoding the specific shRNA for ETK for 96 h and apoptosis was evaluated by TUNEL assays. Apoptotic cells were quantified by counting TUNEL-positive cells from five random independent fields. **B**, ETK regulates ROS activity and cytotoxicity in bladder cancer cells in response to Doxorubucin (DOX) treatment. Cells were infected with lentivirus encoding the shRNA specific for ETK, Etk T7-tagged ETK or a vector control. At 48 h postinfection, cells were treated with DOX (UM-UC-3, 0.5 µg/ml; T24 cells, 2.5 µg/ml) for 20 h and then incubated with 10 nM 5(6)-CDCFDA for 30 min. ROS-positive cells were counted under the fluorescence microscopy. The results were expressed as percentage of the control. **C**, ETK regulates cytotoxicity in bladder cancer cells in response to DOX treatment. Cells were infected as described in B and treated with DOX (T24, 5 µg/ml; UM-UC-3, 0.5 µg/ml) for 48 h. Cell viability was assessed by WST-1 assays (Top panel). The level of ETK expression was monitored by immunoblotting with anti-ETK (Bottom panel). *, p<0.05 compared with the control; **, p<0.01 compared with the control. **D**, Knockdown of ETK inhibited migration and invasion in vitro. Cell migration and invasion assay were described as Methods. The results were expressed as percentage of the control. *, p<0.05 compared with the control; **, p<0.01 compared with the control.

When we examined cellular localization of ETK in bladder cancer cells, we observed a puncate distribution of ETK in the cytoplasm. Interestingly, ETK staining was partially overlapped with Mitotracker labeling in these cells (Fig. 2A). We also detected a significant amount of ETK protein in the mitochondrial fraction, along with other known mitochondrial proteins Bcl-XL and VDAC (Fig. 2B). As ETK lacks a mitochondria targeting signal, we wondered whether it could localize to mitochondria through a protein-protein interaction. As shown in Fig. 2C, endogenous Bcl-XL was co-precipitated with ETK in both bladder cancer cell lines examined. Similar results were also obtained in 293T cells overexpressing both T7-tagged ETK and GFP-tagged Bcl-XL. These data suggested that ETK may form a complex with Bcl-XL and localize to mitochondria. Due to Bcl-XL's anti-apoptotic function, we examined the role of ETK in regulating apoptosis in these cells. As shown in Fig. 3A, knock-down of ETK expression by a specific shRNA significantly increased the number of apoptotic cells in UM-UC-3 and T24 cells. In addition, inhibition of ETK activity enhanced ROS production and cytotoxicity in bladder cancer cells in response to treatment of Doxorubucin, while overexpression of ETK had protective effects (Fig. 3B & 3C). Tumor growth and metastasis is regulated in part by the ability of tumor cells to degrade surrounding matrix tissue and migrate. To test whether ETK upregulation may cause such a phenotype, we further examined the effects of ETK knock-down on bladder cancer cell migration and invasion using the Boyden chamber assays. Fig. 3D shows that migration of ETK-knockdown UM-UC-3 and T24 cells was reduced to 40% and 60% respectively compared with the control shRNA treated cells. Similar results were also observed when we examined their ability to invade through matrigel. These data suggested that inhibition of ETK expression in bladder cancer cells compromised both migration and invasion.

### Etk is upregulated in human bladder cancer tissue and predicted patient survival

**Figure 4:**
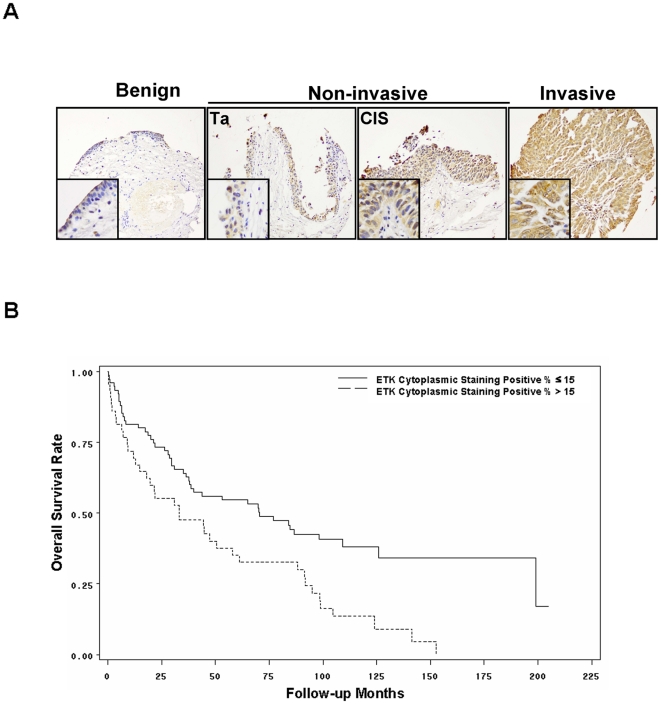
**A**, The representative fields of human bladder cancer TMAs stained with anti-ETK antibody. **B**, Kaplan-Meier analysis on association of ETK cytoplasmic staining with the survival rate of patients with cystectomy.

To examine ETK expression in human bladder tumor tissues, we performed immunohistochemistry analysis on human bladder tissue microarrays containing 619 tissue samples from 233 patients. ETK staining appeared to be both cytoplasmic and nuclear positive (Fig. 4A), and ETK expression was markedly increased in bladder tumor tissues compared to their benign counterparts (Table 1). In addition, ETK expression level was significantly higher in invasive T1-T4 tumors than that in non-invasive Tis/Ta counterparts (p<0.001); however, ETK expression did not differ significantly within T1 to T4 tumors (data not shown). In addition, ETK expression increased with tumor grade (p<0.001). ETK expression in CIS was a notable exception, with a mean 12% in cytoplasm, which is lower than G1–G3 scores (Table 1), suggesting an association of ETK overepression with bladder papillary tumors. To assess whether ETK could be used as a potential prognostic marker, clinical outcome analysis was performed on 118 patients underwent cystectomy who were followed up for a median of 92.3 months. We identified a cutoff point of 15% for optimal substratification of these patients according to ETK cytoplasmic expression. There were 75 tumors (64%) with low expression (≤15%) and 43 tumors (36%) with high expression (>15%). Kaplan-Meier analysis indicated that patients who had higher cytoplasmic staining of ETK (staining score >15%) had poorer overall survival probability (Fig. 4B, log-rank test p = 0.0028). Furthermore, multivariable Cox Regression Analysis showed that ETK is a significant predictor of survival after adjusting for other important clinicopathological variables including age, tumor grade, stage and positive lymph node status (Table 2). As indicated in the model, ETK was the only independent prognostic predictor for overall survival with hazard ratio of 1.7 (95% CI: 1.1 to 2.7, p = 0.0159). Taken together, these data showed that ETK expression is increased in bladder cancer and associated with tumor progression and poor prognosis.

**Table 1:** ETK cytoplasmic expression in bladder tissue.

Group	Mean ± SE	N(%)
**Grade**		
Normal	12.66±0.74	193 (32.60)
G1	24.42±1.82	26 (4.39)
G2	25.18±1.26	125 (21.12)
G3	25.55±0.91	176 (29.73)
CIS	12.17±1.05	72 (12.16)
p Value	<0.0001	
**Stage**		
Normal	12.66±0.73	193 (31.2)
Non-invasive(Tis,Ta)	20.49±0.99	187 (30.2)
Invasive(T1–T4)	25.06±0.82	239 (38.6)
p Value	<0.0001	

**Table 2:** Multivariate Cox regression analysis on survival in patients with Cystectomy.

Covariate	HR (95% CI)	p Value
Age≥70 yr	1.512 (0.968–2.361)	0.0692
Grade 3 vs 1–2	1.264 (0.635–2.514)	0.5045
Stage T1-4 vs Tis/Ta	1.167 (0.480–2.837)	0.7325
N+ vs N0	1.650 (0.934–2.913)	0.0845
**Etk Cytoplasmic (>15)**	**1.732 (1.108–2.706)**	**0.0159**

## Discussion

Overexpression of ETK has been reported in several cancers, including prostate, breast and lung cancers [8], [13], [17], [18]. This is the first report on deregulation of ETK expression in bladder cancer. Frequently elevated ETK expression in bladder cancer cells suggested that ETK may play a causal role in disease development and progression. This was supported by our observation that ETK activity could be stimulated by EGF, which was previously demonstrated to induce growth and enhance invasion of bladder cancer cells by regulating of AKT activity [19], [20]. The EGF receptor has been reported highly expressed in bladder cancer cells and is associated with cancer progression and poor prognosis in patients [21], [22], [23]. Therefore, ETK may likely act as a downstream effector of EGF/EGFR to promote bladder tumor growth and metastasis. It has been shown that ETK overexpression can increase proliferation in mouse prostate epithelium and result in development of prostatic intraepithelial neoplasia (PIN) partly by increasing AKT and STAT3 activity [13]. We found that down-regulation of ETK in bladder cancer cells attenuated STAT3 and AKT activity whereas exogenous overexpression of ETK had opposite effects. Deregulation of STAT3 and PI3K/AKT pathways has been shown to play an important role in the development of urothelial carcinoma and correlates with tumor progression [24], [25]. It is possible that ETK may exert its role in bladder cancer through regulating these pathways. Tumor metastasis is dependent on the ability of tumor cells to invade normal tissues. We showed that bladder cancer cells could effectively invade a matrix barrier in vitro, and this ability was markedly dismissed when ETK expression was knocked down by a specific shRNA. Knockdown ETK leads to diminished activation of STAT3, which plays a role in bladder cancer invasion, partially explained the possible mechanism of invasion inhibition. The effect of ETK on cell migration could be explained based on the established role of ETK in cell migration in prostate and breast cancer cells through FAK-mediated integrin signaling [8].

**Figure 5:**
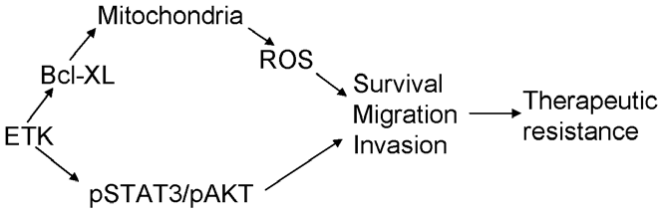
Postulated role of ETK in bladder cancer.

In addition to STAT3 and AKT pathways which are known to be activated by ETK, we also showed, for the first time, that ETK localizes to mitochondria in bladder cancer cells and thereby regulates ROS production and drug sensitivity. Other tyrosine kinases, including Src and EGFR, have been shown to be present in mitochondria. Salvi *et al* reported that Src is located in mitochondria in rat brain [26]. EGFR and Src can translocate to mitochondria by binding the subunit 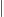 of mitochondrial cytochrome coxidase (Coxα) in an EGF dependent manner and modulate mitochondrial function through phosphorylating Coxα [27]. However the exact function of ETK in mitochondria has yet to be established. We found that ETK is associated with a Bcl-2 family member Bcl-XL in bladder cancer cells. Knockdown of ETK expression leads to an increase of ROS production and sensitization of bladder caner cell to chemotherapeutic drugs. It has been shown that DNA damage, irradiation and hypoxia can induce ROS in cancer cells [28], [29]. We previously showed that ETK can confer drug resistance in prostate cancer cells by interacting with p53 and inhibiting its nuclear transduction function. It is possible that ETK may also promote drug resistance through its protective effect on mitochondrial function. Our results therefore demonstrated that ETK affords a malignant and invasive phenotype to bladder cancer cells through regulating multiple signaling pathways (Fig. 5).

It has been shown that STAT3 activity and expression is increased in bladder cancer tumor and predicts tumor recurrence and patient survival [30], [31]. Our IHC analysis on bladder cancer TMAs showed that ETK expression is increased in bladder cancer tissues compared with their benign counterparts. Considering that targeted expression of ETK in mouse prostate leads to the development of PIN [13], it is possible that ETK may also play a role in oncogenic transformation in bladder urothelial cells. More importantly, ETK expression is higher in invasive (T1–T4) than non-invasive bladder tumors (Tis/Ta), suggesting that ETK may also play a role in tumor invasion and metastasis. This possibility was supported by our observation that knockdown of ETK expression in bladder cancer cells inhibit their activity in *in vitro* invasion assays. Furthermore, we found that ETK expression level also can predict survival of patients with cystectomy treatment independent of other important clinicopathological variables including age, tumor grade, stage and positive lymph node status. Therefore, ETK may potentially serve as a new drug target for bladder cancer treatment as well as a biomarker which could be used to stratify patients with higher mortality risk. These patients may be beneficial from therapeutics targeting ETK activity.

## Materials and Methods

### Cell Culture, transfection and lentiviral infection

Human bladder cancer cell line T24, 5637, J82, RT-4 and UM-UC-3 as well as 293T were purchased from American Type Culture Collection (ATCC). UM-UC-3, T24 and 293T cells were grown in Dulbecco's modified Eagle's medium. 5637, J82 and RT-4 cells were maintained in RPMI 1640 with 10% fetal bovine serum and 1% (v/v) penicillin and streptomycin (100 µg/ml) and maintained at 37°C in a 5% CO_2_ atmosphere. Cells were transfected with HD FuGENE 6 (Roche Molecular Biochemicals) following the manufacturer's instruction. Lentivirus infection was carried out as previously [9].

### Immunoprecipitation and Western Blot

Cells were lysed in the lysis buffer (20 mM Tris/HCl, pH 7.4, 150 mM NaCl, 1 mM EDTA, 1 mM EGTA, 1% Triton X-100, 1 mM Na_3_VO_4_, 1 mg/ml aprotinin, 1 mg/ml leupeptin, and 1 mM phenylmethyl-sulfonyl fluoride). Insoluble material was removed by centrifugation, and antibodies were added to lysate for overnight at 4°C. Antibodies were collected using protein A or protein G-Sepharose beads, and protein complexes were washed three times at 4°C with the lysis buffer. Immunoblotting was done as described previously[9]. Briefly, the equal amounts of sample were resolved on a SDS polyacrylamide gel and transferred to a polyvinylidene difluoride membrane. Blots were incubated with the indicated primary antibodies overnight at 4°C and followed by detection with horseradish peroxidase-conjugated secondary antibody. The monoclonal anti-BMX antibody (BD Transduction Laboratories) was used at 1∶2000, whereas anti-pSTAT3(Y705), AKT, pAKT(S473), EGFR, pEGFR(Y1175) and pETK(Y40) (Cell Signaling Technology, Danvers, MA) were used at 1∶1,000. Anti-Bcl-XL, anti-tubulin, ERK, VDAC and anti-signal transducers and activators of transcription 3(STAT3) (Santa Cruz Biotechnology, Santa Cruz, CA) were used at 1∶2,000.

### WST-1, colony formation and TUNEL assays

Cells were seeded in 96-well plates at a density of 1×10^3^ cell/well and infected with the lentivirus encoding the ETK shRNA or a control shRNA. At each indicated time point, cell viability was measured by WST-1 (Roche) assay following the manufacturer's protocol. For colony formation assay, cells (1×10^4^) were seeded in 6-well plates and infected with the lentivirus for control shRNA or ETK shRNA. Cell culture was maintained in complete medium for 14 days. Cell colonies were then visualized by Coomassie blue staining. UM-UC-3 and T24 cells were infected with the lentivirus encoding ETK shRNA or a control for 96 h and apoptotic cells were detected by TUNEL assay following the manufacturer's instructions (Roche). Apoptotic cells were quantified by counting TUNEL-positive cells from five random independent fields.

### Cell migration and invasion assays

Cell migration was assessed by transwell assay as described previously [8]. Briefly, infected cells were harvested, resuspended in serum-free medium, and then transferred to the top chambers (5×10^4^ cells per well) while the bottom chambers containing 0.5 ml of Conditioned medium from NIH 3T3 fibroblasts. After 24 h incubation, cells on the upper surface were scraped and washed away, whereas the cells migrated to the lower surface were fixed and stained with 4′,6-diamidino- 2-phenylindole (DAPI) for 5 min and then counted under a fluorescence microscope, and the relative number to the vector control was calculated. Cell invasion assay was performed in conditions similarly as above, except for the transwell filters were pre-coated with Matrigel (BD Biosciences) and 1×10^5^ cells were used per well.

### Immunofluorescence and confocal microscopy analysis

Cells were labeled in fresh culture media with 200 nM Mitotracker (Invitrogen) at 37°C for 30 min, and were then fixed with 3.75% formalin for 15 min followed by incubation with blocking solution [10% donkey serum 0.5% bovine serum albumin, 0.3% triton X-100 in PBS] and treated with ETK antibody for overnight at 4°C. The immunoreactions were revealed by incubation of the cells with goat anti-mouse FITC 488–conjugated IgG (Jackson Immuno Research) and DAPI (1∶5000) for 5 min. Finally, the coverslip containing the immunolabeled cells were mounted with an anti-fading mounting medium (Biomeda gel mount, Electron Microscopy Sciences). The cells were evaluated using a water immersion 40X objective lens of a Zeiss 510 confocal microscope equipped with 347-, 488-, and 543-nm laser beams.

### Mitochondrial preparation and Reactive Oxygen Species (ROS) detection

Mitochondria fraction was purified with Mitochondria Isolation Kit for Cultured Cells (PIERCE) according to the manufacturer's instructions. Intracellular ROS was measured by using the dye 5(6)-CDCFDA (molecular probes). After passing through the plasma membrane, this lipophilic and no-fluorescent compound is deesterified to a hydrophilic alcohol (H2DCF) that may be oxidized to fluorescent DCF by a process usually considered to involve with ROS. Cultured cells were loaded with 10 µM 5(6)-CDCFDA in normal medium at 37°C for 30 min. After incubation, cells were washed three times with medium and left the last washing medium for imaging studies. Imaging was taken by fluorescence microscope using a Nikon TE2000s inverted microscope with 10x objective.

### Tissue microarray, Immunohistochemistry and Statistical analysis

Tissue microarrays (TMAs) were prepared by the department of Pathology at the University of California, Los Angeles (UCLA) Medical Center as previously described [14]. Investigators were only provided with clinical-pathological data such as the tumor stage and grade. All patient identifiers were removed so that it is not possible to trace any tissue to a particular patient. Therefore, patient confidentiality is protected. Under the protocol approved by the IRB committee at UCLA, no patient consent is required for this study. TMA were deparaffinized with xylene and stained as previously described [13]. Briefly, slides were rehydrated and antigen retrieval was achieved by microwave for 15 min in citrate buffer. Slides were then incubated in 0.3% hydrogen peroxide to quench endogenous horseradish peroxidase (HRP) for 30 min. Slides were preincubated with normal goat serum in PBS (1∶20) for 60 min at room temperature. Slides were then incubated with primary antibody ETK (1∶2000) at 4 degree overnight. Subsequently, slides were incubated with biotin-labeled anti-rabbit IgG and incubated with preformed avidin-biotin peroxidase complex. Finally, the slides were counterstained with hematoxylin, dehydrated and mounted.

Slides were analyzed using the Ariol SL-50 automated slide scanner (Applied Imaging, San Jose, Calif) to quantitate the amount of positive staining for each area of interest. Thresholds for each image were applied using the Ariol analytical software based on multiple parameters: RGB algorithm, shape and size. All analyses were performed with the MultiStain script. Thresholded classifiers were customized for each stain.

For assessing nuclear staining, positive DAB staining was calculated by applying two color thresholds with one recognizing blue background (haematoxylin stained) cells and another recognizing brown positive cells and blue, non-positive cells (total cell number). Individual cells were discriminated by incorporating the shape and size thresholds, providing, together with the color thresholds, actual cell counts. Percent of positivity was determined by dividing the cell number detected by the brown threshold by the total cell number, detected by the sum of the brown and blue thresholds. Total tissue area analyzed was also included in the final analysis (µm^2^).

For assessing cytoplasmic staining, the area positive stain was calculated by applying color thresholds to detect positive brown pixels. Percent of positivity was determined by dividing the total positive stain area (µm^2^) by the total tissue area analyzed (µm^2^).

The immunoreactive score for each case was quantified by the average of four cores. Associations between ETK expression and pathological parameters were assessed with the nonparametric Kruskal-Wallis tests. Different survival/recurrence endpoints were assessed for patients who underwent TUR and patients who underwent cystectomy. Kaplan–Meier curves were used to estimate survival/recurrence-free time curves and the log rank test was used to test whether the curves differed between groups. COX multiple proportional hazards model was used to assess which covariates affect survival/recurrence-free time. For each covariate, the relative hazard rate and the associated *P* value were reported. All analyses were performed with the software SAS version 9.2.
